# Healable Carbon Fiber-Reinforced Epoxy/Cyclic Olefin Copolymer Composites

**DOI:** 10.3390/ma13092165

**Published:** 2020-05-07

**Authors:** Haroon Mahmood, Andrea Dorigato, Alessandro Pegoretti

**Affiliations:** 1 Department of Industrial Engineering, University of Trento, via Sommarive 9, 38123 Trento, Italy; andrea.dorigato@unitn.it; 2 National Interuniversity Consortium of Materials Science and Technology (INSTM), via Giuseppe Giusti 9, 50121 Florence, Italy

**Keywords:** carbon fibers, laminates, multifunctional composites, fracture toughness

## Abstract

Cyclic olefin copolymer (COC) particles were dispersed in various amounts in an epoxy matrix, and the resulting blends were used to impregnate unidirectional carbon fibers (CF) by hand lay-up. The thermal stability was not substantially modified by the presence of COC particles. The mixture of the two polymers resulted in a phase separated blend and the flexural modulus and interlaminar shear strength progressively decreased with the addition of COC particles in the laminates. Mode I fracture toughness tests were executed on double cantilever beam specimens. The opened crack was then thermally mended at 190 °C for 1 h. The laminates containing 30 wt.% of COC particles showed a healing efficiency of ~180%.

## 1. Introduction

In recent decades, polymer composites were introduced in an increasing number of applications because of their peculiar combination of properties, not otherwise attainable from conventional materials like neat polymers, metals or ceramics. For example, carbon fiber reinforced composites are extensively applied in the new generation of commercial aircrafts, like Airbus A350 XWB (53 wt.%) and Boeing 787 Dreamliner (50 wt.%). A strong interest has recently arisen around the possibility to further promote the reliability and the safety of composites by introducing structural health monitoring [1,2,3,4,5] and self-healing capabilities [6,7,8,9].

In recent years, a strong interest arose for self-healing thermosetting materials and many efforts have been made to induce self-healing capabilities to thermosetting resins in such a way that any damage or defect could be healed [10]. Generally, the self-healing phenomenon of polymeric materials can be classified as extrinsic or intrinsic. Extrinsic systems consist of matrices filled with auxiliary components like capsules or vascular networks. Such type of matrices has been used in fiber reinforced composites, but they demonstrated poor healing capabilities and substantial losses in structural properties [11,12]. On the other hand, intrinsic self-healing systems are based on specific molecular structures that may induce crack healing with the need of external stimuli (such as heat, light, UV etc.). Self-healing polymeric systems have been extensively studied in recent years, either in the form of resin or when reinforced with fibers [13,14,15,16]. In these systems, a semicrystalline thermoplastic polymer (having low melting point) is added in the thermosetting resin, and a miscible [9] or immiscible blend [17,18,19,20] can be thus created. In addition to these, particulate composites where particles of poly(ethylene-co-(methacrylic acid)) (EMAA) [21] or copolyester [22] were dispersed as healing agents in polymer matrices have been also reported.

Extrinsic systems for self-healing in fiber reinforced polymer composites have been widely investigated in the past. Patel et al. [23] studied self-healing of impact damage in glass-fiber-reinforced epoxy composites containing microencapsulated dicyclopentadiene (DCPD) liquid healing agent. However, the presence of wax-encapsulated particles imparted longer crack lengths in the laminates when tested for improving compression after impact (CAI) strength [23]. The author reported a maximum healing efficiency of 95%. The work of Yin et al. [24,25,26] reported in detail the use of epoxy loaded with capsules in fiber-reinforced polymer composites in which a copper-based catalyst was embedded. Although the fiber content was less than 30%, the maximum healing efficiency reported was over 70% in the case of 30% capsules-loaded epoxy and 2% latent hardener (i.e., CuBr_2_(2-MeIm)_4_). The work of Bolimowski et al. [27] also showed the use of epoxy resin loaded with microcapsules along with particles of scandium (III) triflate (as a catalyst) in the interlaminar region of unidirectional carbon fiber (CF)-reinforced polymer laminates. The opening mode fracture toughness test performed on the specimens caused the laminates to crack the resin filled with microcapsules, hence releasing the epoxy resin to react with the catalyst to heal the fracture surface. The authors reported a maximum recovery of 44% in mode I fracture toughness in the composites. As a drawback, for these composites, a decrease in the initial strain energy release rate (G_Ic_) was observed. Other than epoxy resin (EP) as healing agent in microcapsules, ethyl phenylacetate (EPA)-filled capsules were also considered by Manfredi et al. [12]. In addition, hollow fibrous structures with healing agents have been proven to show a healing efficiency of up to 84% in fiber-reinforced polymer composites [6,28,29,30,31].

Intrinsic self-healing materials do not have a sequestered healing agent but instead have a latent self-healing functionality that is triggered by damage or by an outside stimulus. Usually, an external stimulus like heat is required to heal the damage and defects in the structure. One possible way to obtain reversibility of the system is by molecular interactions within the polymer matrix, such as the reversible Diels–Alder reaction. A thermally mendable polymer called Mendomer 401 was investigated for the first time in carbon fiber laminates by Park et al. [32]. They demonstrated the repairability of the system by three-point bending tests on laminates consisting of two layers of carbon fiber fabrics and healing efficiency values between 92–94% were obtained. In a similar work of Park et al. [33], a bismaleimide tetrafuran (2MEP4F) was used to thermally mend carbon fiber reinforced composites through a Diels–Alder reaction. A healing efficiency of more than 90% was reported upon a thermal treatment on the damaged specimens lasting longer than 3 h. Using short beam shear tests, the evaluation of healing by Diels–Alder reaction in the carbon fiber laminates was averaged at 85% [34]. Other than Diels–Alder reaction, exchangeable disulfide crosslinks were also used by Luzuriaga et al. [35], and a healing efficiency of about 100% was obtained in the repaired specimens, as assessed by short beam shear tests. The relatively new technique of supramolecular assembly has been taken in consideration for the healing of thermosetting polymers, though the majority of the work has been performed on nonreinforced polymers [36,37,38]. Moreover, the only application of supramolecular assembly for self-healing in glass fiber-reinforced composites is reported in the work of Sordo and Michaud [39]. The healing was performed by a prepolymer blend of poly carboxylic fatty acids reacting with H-bonding heterocyclic primary amine (UDETA) and diepoxide (DGEBA). A maximum recovery degree of 72% for the elastic modulus and of 65% for the flexural strength was reached.

The concept of using a thermoplastic–thermosetting blend as a matrix in fiber-reinforced composites has received some attention in the last years. In these systems, the dispersed thermoplastic phase could close the cracks hence performing the healing of the laminates containing defects. The pioneering work in this area was reported by Hayes et al. [9,40] in 2007, in which a thermoplastic polymer (polybisphenol-A-co-epichlorohydrin) was dissolved in an epoxy resin to create a miscible blend that was used as a matrix for glass fiber-reinforced laminates. Healing was performed by heating the damaged samples at 130 °C, in such a way that the thermoplastic phase could flow through the epoxy and mend the damaged areas. However, no quantitative calculation of the mechanical healing was reported in this paper. The use of immiscible blends for thermal mending has also been reported by Luo et al. [18]. The authors used an epoxy/poly(ε-caprolactone) (PCL) blend in which an induced phase separation of PCL in epoxy matrix resulted in a “brick and mortar” structure capable of healing the specimens, and the load bearing capacity of healed samples surpassed that of the virgin specimens. Other immiscible blends with different polymers have been reported in the literature [7,17,20,41,42]. Other techniques used in the past to create mendable systems include the use of thermoplastic films or woven fabrics in the laminates [41,43,44,45]. In these works, healing efficiency levels of around 100% were obtained, depending on the placement of the healing agent within the system. Further work was performed by the group of Pingkarawat et al. [46,47,48] on CF laminates, with healing efficiency in terms of fracture toughness of around 300%, even if the in-plane properties after healing were low.

Recently, Mahmood et al. explored the use of cyclic olefin copolymers (COCs) as an amorphous thermoplastic healing agent in an epoxy matrix [8]. COCs are amorphous thermoplastic polymers with mechanical properties comparable with those of epoxy resins, and are available in various grades with different glass-transition temperatures [49,50]. The healing efficiency detected on SENB specimens was around 100% when 40 wt.% of COC in particle form was dispersed in the epoxy matrix. Starting from that results, the present paper investigates the healing potential of EP/COC blends in carbon fiber-reinforced composites. The blends will be applied in the preparation of unidirectional CF laminates through a simple hand lay-up technique. The fracture behavior of virgin and healed composites will be investigated under quasi-static conditions, in order to evaluate the thermal mending capability of the prepared blends.

## 2. Materials and Methods

A bicomponent epoxy system (EP) consisting of an epoxy base (EC157) and an aminic hardener (W 342) was kindly provided by Elantas Europe Srl (Collecchio, Italy). After a recommended curing cycle of 24 h at room temperature followed by 15 h at 60 °C, the resin manifested a glass transition temperature (T_g_) of 85.4 °C [51]. COC granules (TOPAS^®^ 8007) were supplied by Ticona (Kelsterbach, Germany). This COC grade contained 65 wt.% of ethylene and 35 wt.% of norbornene (melt flow index at 190 °C and 2.16 kg = 1.7 g/10 min, density = 1.02 g/cm^3^, T_g_ = 78 °C). Unidirectional carbon fibers fabrics (GV-201 TFX), having a nominal surface density of 218 g/m^2^, were purchased by Angeloni Srl (Quarto d’Altino, Venice, Italy) and were used as reinforcement. All the materials in this work were used as received, without any further treatment.

COC granules were cryogenically milled in liquid nitrogen by using an IKA^®^ Labortechnik M 20 grinding machine. The grinded powder was sieved by a 300 µm mesh and added at different concentrations to the epoxy base (10, 20 and 30 wt.%) at 50 °C and 3000 rpm for 1 h. Degassing was performed on the solution for 15 min to remove mixed air. The hardener was added at a base/hardener relative ratio of 100/30 and degassed again for 2 min. CF fabrics were uniformly impregnated with the prepared blends and stacked over each other by hand lay-up method using a roller. Consolidation of stacked impregnated fabrics was performed through a vacuum bag placed in a hot press, applying a pressure of 0.8 MPa. Thermal curing in the hot press was performed according to the technical data sheet of the producer of the resin (i.e., 24 h at 23 °C + 15 h at 60 °C). In this way, unidirectional epoxy/CF laminates and composites with different amounts of COC (10, 20, 30 wt.%) in the matrix were prepared. The prepared samples were designated as EP/CF for the composite without COC (control sample), or as EPxCOC/CF for the composites with COC as healing agent (x = 10–30 wt.%).

The microstructure of the EP/CF and EP-COC/CF composites was analyzed by using a Zeiss Axiophot optical microscope (Oberkochen, Germany), coupled with a Leica DC300 digital camera (Leica Microsystems Ltd., Heerbrugg, Switzerland). The specimens were polished using abrasive grinding papers with grit size P800, P1200, and P4000, sequentially.

Thermogravimetric analyses (TGA) were performed through a Mettler TG50 (Schwerzenbach, Switzerland) machine. Tests were carried out between 35 °C and 700 °C, at a heating rate of 10 °C·min^−1^ and under a constant nitrogen flow of 100 mL·min^−1^. On the obtained thermograms, the temperatures associated with the onset degradation (Tonset) and the decomposition of epoxy matrix (TdEP) and COC (TdCOC) were evaluated. In addition, the residual mass (in percentage) at 700 °C (m700), corresponding to the real weight percent of the CF present in the composite, was determined.

The density of the neat epoxy matrix and of the composites (ρ_exp_) at different COC amounts was measured at 23 °C by using a Gibertini E42 (Modena, Italy) precision balance, having a sensitivity of 10^−4^ g. According to the ASTM D792 standard, the specimens were weighed in air and in ethanol, and the density was thus calculated.

The density of the CF was measured employing a Micromeritics^®^ Accupyc 1330 helium pycnometer (Micromeritics Instrument Corporation, Norcross, GA, USA) at 23 °C, by using a testing chamber of 3.5 cm^3^. The fiber volume fraction (*V_f_*) in the composites was calculated by using the expression reported in Equation (1):

(1) $$V_{f}=\frac{1}{1+\frac{\rho_{f}}{\rho_{m}}\left(\frac{1}{W_{f}}-1\right)}$$

where *ρ_f_* and *ρ_m_* are respectively the density of the fiber and matrix, while *W_f_* is the fiber weight fraction. The theoretical density (*ρ_t_*) of the composite specimens was then estimated by using Equation (2):

(2) $$\rho_{t}=\rho_{f}\cdot V_{f}+\rho_{m}\cdot V_{m}$$

where *V_m_* represents the matrix volume fraction. It is possible to also estimate the volume fraction of the voids (*V_v_*) in the specimen using Equation (3):

(3) $$V_{v}=\frac{\rho_{t}-\rho_{exp}}{\rho_{t}}$$

Flexural properties of the prepared laminates (containing 4 layers of CF fabric) were determined by using an Instron^®^ 5696 universal testing machine (Norwood, MA, USA), following the ASTM D790 standard. Rectangular specimens with dimension having a mean width of 12.7 mm were tested imposing a span to depth ratio of 60:1 and 40:1 for the measurement of flexural modulus and of the flexural strength, respectively. The tests were performed imposing a strain rate in the outer fiber of 0.01 mm^−1^. At least five specimens were tested for each sample.

Interlaminar shear strength (*ILSS*) values of the composites (having 16 CF fabric layers) were determined by short beam shear (SBS) test, performed according to the ASTM D2344 standard. Testing was performed under 3-point bending configuration by using an Instron^®^ 5969 tensile testing machine at a cross-head speed of 1 mm min^−1^. The *ILSS* was calculated by using the maximum load sustained by the samples (*F_max_*), applying the expression reported in Equation (4):

(4) $$ILSS=0.75\times \frac{F_{max}}{b\times h}$$

where *b* and *h* are the width and thickness of the specimens.

**Figure 1:**
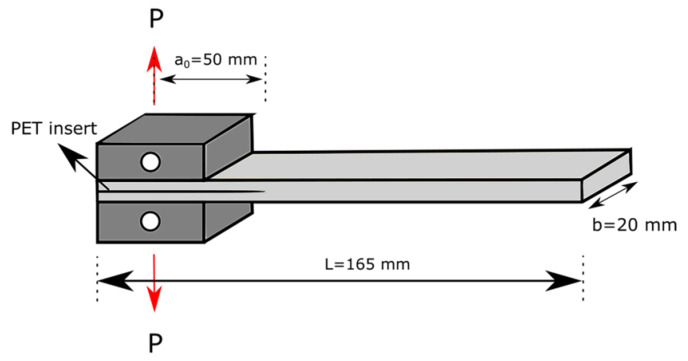
Dual cantilever beam (DCB) specimen for Mode I fracture toughness test.

**Figure 2:**
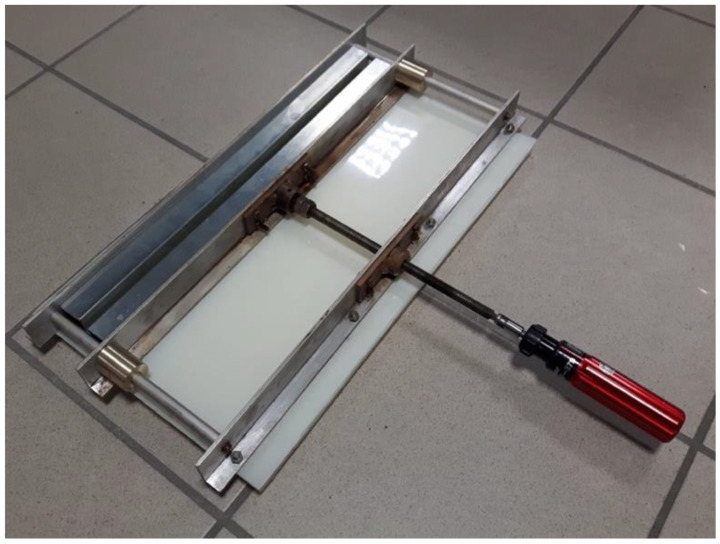
Screw driven mold used for healing along with the torque wrench.

The evaluation of the fracture behavior of the prepared laminates (containing 14 CF fabric layers) was performed adopting the procedure indicated in the ASTM D5528 standard. Dual cantilever beam (DCB) specimens 165 mm long and 20 mm wide, as shown in Figure 1, were prepared by inserting a thin (26 µm) polyethylene terephthalate (PET) foil in the middle of the laminate to obtain a precrack of at least 65 mm. Loading blocks were attached to the composite specimen in such a way that the load applied to laminate to open the crack was 50 mm away from the crack tip. The crack advancement monitoring during the test was performed by using a digital webcam (Logitech^®^ B910HD) that recorded the video in synchronization with the loading test. The specimens were tested by using an Instron^®^ 5969 machine at a crosshead speed of 2.5 mm·min^−1^. Prior to testing, the specimens were conditioned at 23 °C for at least one hour. The specimens were precracked by loading them until (on average) 5 mm of crack advancement, followed by unloading and reloading for fracture toughness testing until a crack advancement of ca. 50 mm. During the tests, the applied load (P), crack opening displacement (*δ*) and crack length (*a*) values were measured simultaneously. By applying Equation (5), the mode I interlaminar fracture toughness (*G_I_*) was calculated for each crack length:

(5) $$G_{I}=\frac{3P\delta }{2b\left(a+\left|\Delta \right|\right)}$$

where *b* is the specimen width and |Δ| is a factor used to correct the vertical displacement and rotation effects at the delamination crack tip. On the basis of the delamination growth, a resistance curve (R curve) was generated to characterize the initiation and propagation of the delamination. For the evaluation of the healing efficiency of the laminates, the initiation values of *G_Ic_* were selected on the basis of (i) nonlinearity in the load–displacement curve (*NL*) and (ii) at the point where delamination was visually observed (*VIS*). In this way, the mode I interlaminar fracture toughness of virgin specimens corresponding to NL and VIS (*G_Icv_^NL^* and *G_Icv_^VIS^,* respectively) were determined. To thermally mend the cracked DCB specimens during the test and evaluate the healing efficiency, a lab-made screw-driven device was used as shown in Figure 2. A torque wrench was used to apply a compressive pressure of 500 kPa. The thermal healing was performed by placing the whole setup in an oven at 190 °C for 1 h.

The repaired specimens were tested again by Mode I fracture toughness test until the delamination was completed on the healed part of the specimen. The *G_Ic_* values corresponding to *NL* and *VIS* (*G_Icr_^NL^* and *G_Icr_^VIS^*, respectively) were determined similarly to that of the virgin specimens. The apparent healing efficiency (*η*) of the prepared samples was thus computed as reported in Equations (6) and (7):

(6) $$\eta_{NL}=\frac{G_{Icr}{}^{NL}}{G_{Icv}{}^{NL}}$$

(7) $$\eta_{VIS}=\frac{G_{Icr}{}^{VIS}}{G_{Icv}{}^{VIS}}$$

where $\eta_{NL}$ refers to healing efficiency calculated from the initiation value of G_Ic_ at NL, while $\eta_{VIS}$ relates healing efficiency computed from *G_Ic_* at VIS.

Analysis of the crack surfaces before and after the healing was carried out through a field emission scanning electron microscopy (FESEM). A Zeiss SUPRA 40 microscope was employed to analyze the specimens, which were coated by a 5 nm thick layer of platinum/palladium alloy (80:20) coating.

## 3. Results

**Figure 3:**
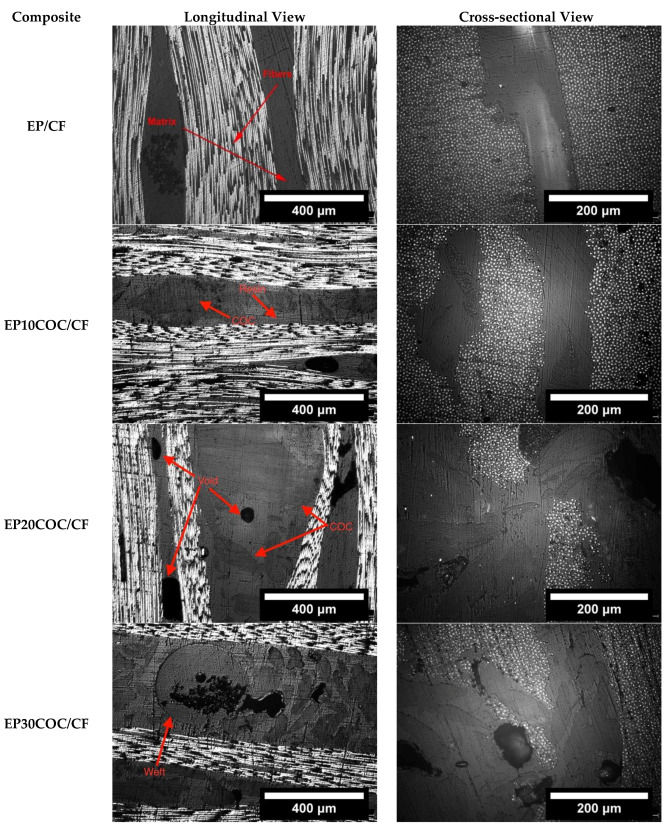
Optical microscope images of epoxy system/carbon fiber (EP/CF) and epoxy system x cyclic olefin copolymers/carbon fiber (EPxCOC/CF) composites (x = 10–30 wt.%).

It is well known that the mechanical properties of composite laminates are strongly connected to their microstructural features. Therefore, optical microscope images of the polished surfaces of the produced laminates were collected. Figure 3 shows the microstructure of the prepared composites with and without COC particles. It can be seen that the COC particles are present in the resin rich area between the plies and have an irregular shape. Even if a sieve with a mesh size of 300 µm was used to remove larger COC particles during the production of the samples, COC domains have a larger size with respect to fibers, and thus they could not fit among the fibers within the fabric and remain preferentially dispersed within the matrix in the interlaminar zone. Moreover, COC particles remain solid during the mixing and the curing operations. This creates a partial disruption of the fabric orientation and also increases the thickness in the laminates containing COC. This phenomenon can be also explained considering that the viscosity of the uncured resin increases with the COC concentration, as recently reported by Mahmood et al. [8]. This can be verified by comparing the optical microscope pictures of EP/CF with that of the laminates containing COC (e.g., 20 wt.% or 30 wt.%), where it can be noticed that the thickness of the resin rich area is higher in the composites with an elevated COC concentration. The increase of the viscosity detected in the matrices at elevated COC concentrations and the relative processability issues could also explain the presence of some voids in the EP20COC/CF and EP30COC/CF samples. Some of these negative issues could be positively solved in the future in attempts to further reduce the COC particles size by using efficient cryogenic machines that would overcome the limitations associated to grounding to smaller sized particles.

**Figure 4:**
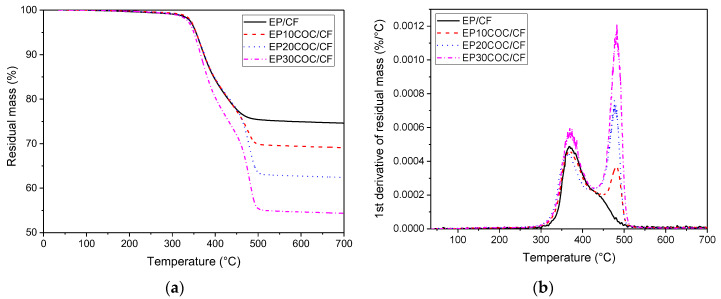
Thermogravimetric analyses (TGA) analysis of EP/CF and EPxCOC/CF composites (x = 10–30 wt.%). (**a**) Residual mass and (**b**) derivative of the mass loss.

TGA analysis was performed to investigate the thermal degradation resistance of the prepared composites. In Figure 4a,b the trends of the residual mass and of the derivative of the mass loss as a function of the testing temperature are respectively reported.

Looking at the Figure 4a, the residual mass curves show that the onset of the degradation process is not substantially influenced by the COC introduction, but the residual mass decreases with the COC content. This indicates that the CF content is lower in the laminates containing COC. This occurred because COC particles remain in the laminate as vacuum and pressure was applied during the production of the samples, however only the liquid resin was squeezed out. Hence, overall, the fiber/matrix ratio decreased due to the increased COC content which sequentially increased the thickness of the laminates. From the derivative thermograms reported in Figure 4b, it is interesting to notice that EP/CF laminate shows a single degradation step at around 370 °C, while the presence of COC in the other laminates can be seen by the presence of another degradation peak at higher temperatures (around 490 °C), whose intensity is proportional to the COC concentration. Table 1 summarizes the data regarding the thermal degradation of the laminates. It can be seen that the introduction of the COC in the laminates do not substantially affect the thermal behavior. In fact, it is worthwhile to note that even at 30 wt.% addition of COC, the structure of the laminate is dominated by the relatively stable CF. In addition, as mentioned before, due to the nature of the fabrication procedure, the residual mass significantly decreased with the introduction of COC particles.

Coupling information deriving from the thermogravimetric analysis and pycnometry measurements, it was possible to determine the volumetric fraction of the constituents and the void concentration in the laminates (see Equations (1)–(3)). These results are collected in Table 2.

As already seen in TGA analysis, the concentration of the CF-reinforcing phase significantly decreases with the COC concentration due to the presence of solid COC particles in the interlaminar region and the viscosity increase at elevated COC amounts. For the same reason, the void content in the laminates containing COC is about two times than that detected for the EP/CF laminate, reaching a value of 12 vol.% for the EP20COC/CF composites. It is therefore clear that a lower concentration of CF in the sample could probably lead to lower mechanical properties, and the increase in the void content could also negatively affect the stress at break.

**Figure 5:**
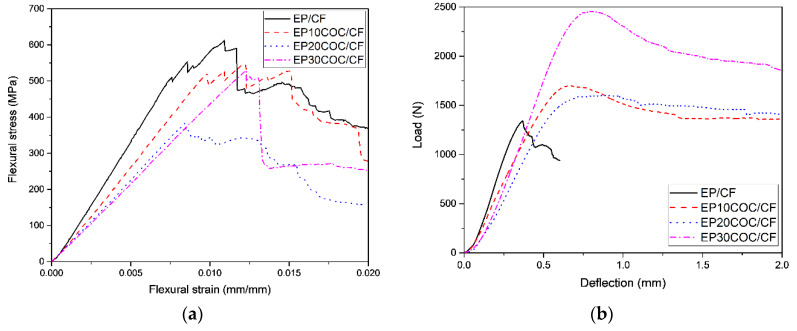
Mechanical tests on the prepared composite laminates. (**a**) Representative flexural stress–strain curves, (**b**) load-deflection curves from short-beam shear tests.

Flexural properties of the prepared composites were evaluated through three-point bending tests. Typical stress–strain curves are shown in Figure 5a while the obtained results are summarized in Table 3.

As expected, the flexural modulus decreases with the COC concentration, passing from 66 GPa of the EF/CF laminate down to 44 GPa of the EP30COC/CF sample. This result could be explained with the observed decrease of the CF content within the laminate. Furthermore, the stress at break is negatively affected by the introduction of COC, even if the drop is not dramatic at elevated COC concentrations. Considering standard deviation values, it can be concluded that flexural strain at break are not negatively affected by COC addition.

In order to evaluate the interlaminar adhesion in the prepared laminates, short beam shear (SBS) tests were performed. Representative load–displacement curves are shown in Figure 5b, while ILSS values are summarized in Table 3. These tests reveal a decrease in interlaminar adhesion when COC is added in the composites. This can be justified by the fact that commercially available CF are normally sized with an epoxy-compatible agent which has a good interfacial adhesion with the epoxy matrix. In case of neat EP/CF composite, the ILSS values are relatively high (i.e., 50.6 MPa), while the introduction of a polymer having a limited wettability with the sized CF leads to an important decrease of the ILSS values (up to 28.8 MPa with a CF content of 20 wt.%). It is also clear that the COC phase is present only in the interlaminar region as solid domains with irregular shape, and this could also explain the observed drop in the interfacial adhesion values.

**Figure 6:**
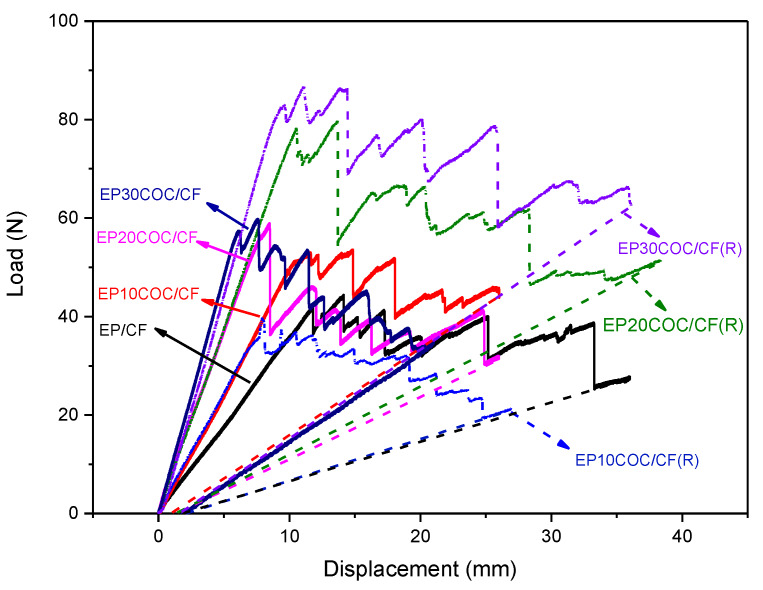
Representative load–displacement curves of Mode I fracture toughness test performed on composite laminates before and after healing (where (R) refers the thermally mended specimens).

The fracture behavior of the prepared laminates was evaluated through G_I_ tests. Representative load–displacement curves of the tested DCB specimens of all composite (virgin and repaired) samples are shown in Figure 6.

Looking at the load-displacement curve of EP/CF sample, it can be seen that the stiffness of the sample is rather low. This could be simply due to the fact the thickness of this laminate is lower than that of the composite with elevated COC amount, and the higher fiber concentration is not able to counterbalance this geometrical effect. The EP/CF do not manifest any repairing capability, while for the repaired sample of composite laminates with COC, the crack propagation behavior is different. The specimens containing COC loading are able to sustain higher loads before crack propagation. This phenomenon is related to the toughening of the laminates due to the presence of a thermoplastic polymer like COC. In addition to this, it can be seen that the load sustained by the healed specimens (except for EP10COC/CF) is considerably higher than that of the corresponding virgin specimens, suggesting a very interesting healing efficiency. This result therefore seems to confirm the elevated healing efficiency values detected in our previous paper on epoxy/COC matrices [8].

Through the comparison between the G_Ic_ values (corresponding to NL and VIS) of the virgin and the repaired specimens, the healing efficiency of the prepared composites was thus determined. The healing efficiency values of the produced laminates are summarized in Table 4. At a first glance, it can be seen that in all cases, the percentages of healing efficiency calculated by G_Ic_ values corresponding to nonlinearity (NL) or visual propagation of the crack (VIS) are nearly similar. In some cases, the scatter of values is very high which could be associated to the (i) misalignment of the crack faces while unloading the sample and/or (ii) the difficulty of determining the advancement of the crack in the healed state [17]. The latter is particularly true (for COC loaded laminates) since the amorphous COC particles above its glass transition temperature diffuses in the crack plane [52], which could generate an incomplete filling of it.

The healing efficiency for EP/CF is rather low, since the crack propagates very easily within the material for very small loads. Thus, it can be considered that a very low healing is achieved by epoxy alone. This limited healing could be associated to the residual cross-linking and/or softening of epoxy at 190 °C during the healing time (1 h). The addition of 10 wt.% of COC in the laminates leads to healing efficiency values of around 60%, meaning that a partial recovery of the original fracture toughness of the laminates occurred. This suggests that the COC particles, softens during the thermal treatment at 190 °C, and act as a hook between the two cantilevers of the DCB specimen during reloading operations, partially sustaining the load applied to the specimens, and thus impeding the crack propagation. By increasing the COC content up to 20 wt.%, a dramatic increase of the healing efficiency can be observed, i.e., an increase of up to ca. 178%, and this value slightly rose up to a range of 180% if the COC content was increased at 30 wt.%. Even with an elevated coefficient of variation is observed, the healing obtained by COC particles in the produced laminates is still impressive. Healing efficiencies associated with the visual crack propagation in Table 4 clearly indicates that the COC particles significantly increased the critical fracture toughness values for the crack propagation, which can be supported by the load–displacement curves shown in Figure 6. Attempts to evaluate the multiple healing efficiencies at optimized thermal mending parameters will be performed in the future.

**Figure 7:**
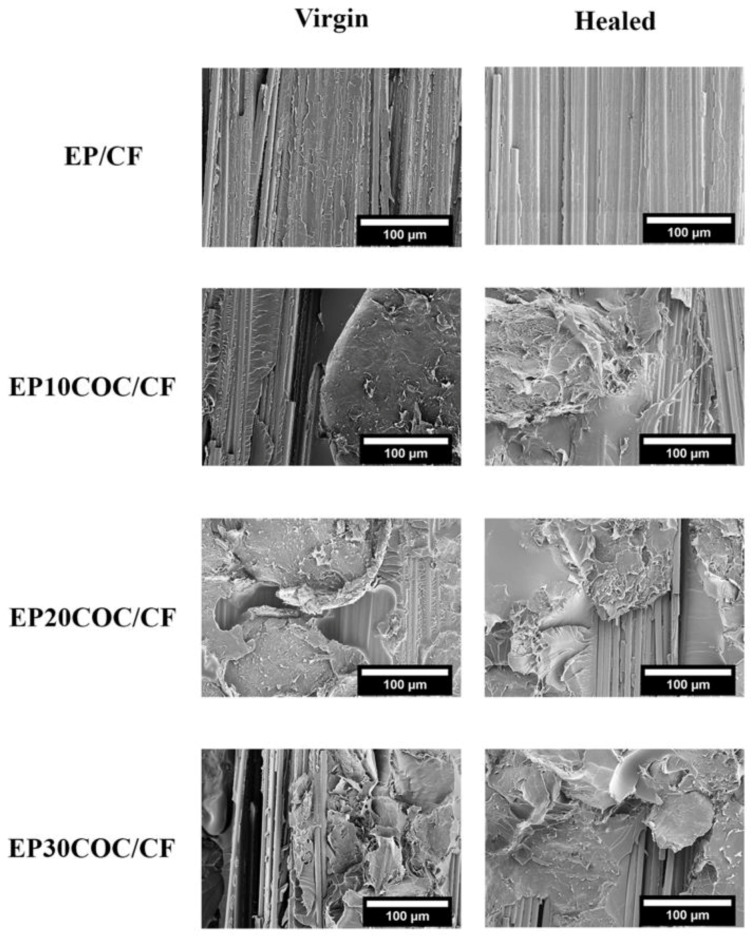
SEM images of Mode I opened DCB specimens before and after healing process.

The healing phenomenon was also investigated by SEM observations of the fracture surfaces of DCB specimens after Mode I opening, and the micrographs are shown in Figure 7.

The column of virgin specimens includes the images of all the virgin specimens that were subjected to Mode I fracture toughness tests and then analyzed under SEM. On the other hand, the column of the healed specimens includes the images of the DCB specimens that were first cracked, then healed and reloaded in the fracture toughness tests. The virgin EP/CF specimen shows a textured microflow pattern which is a characteristic feature of the fracture surface of epoxy under opening mode I [53]. However, the healed version of the same composite shows a relatively smooth fracture surface due to the pertaining healing conditions, which cause softening of the epoxy and flattening of the surface while in compression. Meanwhile, the images of samples containing COC show the presence of large size particles on the surface, whose concentration increases with the COC amount. Before healing, COC domains do not show any fracture propagation profile on their surface, suggesting that the propagating crack did not penetrate the COC, but rather, it traveled on the surface of COC. Only in the EP30COC/CF laminate the crack is able to penetrate within the COC particles.

Looking at the images of the healed specimen, the same particles show a mixed mode fracture surface. In these samples, the COC particles show dimples on their surface, which is the typical fracture profile of ductile material under slow crack propagation conditions. Moreover, some mirror-smooth fracture surfaces on the same particles are also visible on the same samples, which could indicate a fast crack propagation stage. Both of these microstructural features can be correlated to the load–displacement curve of the repaired laminates as shown in Figure 6, in which slow crack propagation stages correspond to the load increase, while the fast crack propagation stages can be associated to the sudden load drop. Such observations confirm the thermal mending effectiveness of the prepared composite structures due to the presence of the amorphous thermoplastic phase in the interlaminar region. It is very difficult, from the experimental point of view, to quantify any difference between the propagation behaviour of the crack in the two phases (epoxy and COC) of the composite matrices. In our previous study [49], we found a fracture toughness value (G_IC_) for this COC grade of about 3 kJ/m^2^. This value is much higher than the fracture toughness value of the epoxy matrix, which was estimated to be about 1 $MPa\sqrt{m}$ [51]. Therefore, we can assume that the presence of COC in the epoxy matrix could play a positive effect on its fracture toughness, thus hindering the crack propagation.

## 4. Conclusions

In this work, amorphous thermoplastic COC particles were dispersed in different amounts in an epoxy matrix, and unidirectional carbon fiber fabrics were impregnated with the prepared blends to obtain novel composite laminates with healing capability. The presence of COC particles in the interlaminar region and the increase in the viscosity of the resin led to a partial distortion of the fabrics, and to a decrease in the relative concentration of carbon fibers within the laminates associated with a substantial porosity increase (up to 10–12 vol.%). Thermal degradation behavior was not substantially affected by the introduction of the COC particles, and the two polymer phases seemed to behave like a phase separated blend with a limited interfacial interaction. Consequently, flexural modulus progressively decreased upon COC addition, and the ILSS values were also considerably reduced. Fracture toughness and thermal mending behavior of the prepared laminates was evaluated through Mode I fracture toughness tests, and COC particles dispersed within the epoxy matrix demonstrated an interesting capability to heal the cracks. With a relative COC amount of 30 wt.%, an overall efficiency of around 180% was achieved. SEM images on the tested DCB specimens showed that the increase in the G_I_ in the healed samples at elevated COC amounts was due to the propagation of the crack within the COC phase with a progressive plasticization of the thermoplastic phase.

## Figures and Tables

**Table 1:** Results of TGA tests on EP-CF and EPxCOC/CF laminates (x = 10–30 wt.%).

Composite	T_onset_ [°C]	T_dEP_ [°C]	T_dCOC_ [°C]	m_700_ [%]
EP/CF	340.8	368.5	-	72.8
EP10COC/CF	339.5	368.9	481.2	66.8
EP20COC/CF	339.6	366.0	479.8	60.6
EP30COC/CF	338.4	369.7	482.0	50.4

**Table 2:** Concentration of the constituents in the prepared composite laminates.

Composite	*W_f_* (wt.%)	*W_EP_* (wt.%)	*W_COC_* (wt.%)	*V_f_* (vol.%)	*V_m_* (vol.%)	*V_v_* (vol.%)
EP/CF	72.8	27.3	0	63.2	31.7	5.1
EP10COC/CF	66.8	25.2	8.0	55.7	34.1	10.2
EP20COC/CF	60.6	22.4	17.0	48.4	39.3	12.2
EP30COC/CF	50.4	25.7	23.9	38.1	52.9	9.0

**Table 3:** Flexural properties and interlaminar shear strength *(ILSS*) values of the prepared composites.

Composite	Flexural Modulus (GPa)	Flexural Strength (MPa)	Flexural Strain at Break (%)	ILSS (MPa)
EP/CF	65.7 ± 5.6	590 ± 28	1.2 ± 0.1	50.6 ± 1.4
EP10COC/CF	46.0 ± 1.9	547 ± 36	1.3 ± 0.1	39.2 ± 1.1
EP20COC/CF	43.8 ± 2.5	388 ± 17	1.1 ± 0.2	28.8 ± 1.2
EP30COC/CF	44.0 ± 3.0	544 ± 59	1.4 ± 0.2	32.6 ± 4.3

**Table 4:** Healing efficiency of laminates calculated by G_Ic_ values corresponding to NL and VIS points.

Composite	Healing Efficiency (%) NL	Healing Efficiency (%) VIS
EP/CF	5.9 ± 3.8	8.6 ± 7.5
EP10COC/CF	64.1 ± 12.2	65.6 ± 11.7
EP20COC/CF	178.5 ± 47.1	179.0 ± 32.8
EP30COC/CF	188.4 ± 57	179.3 ± 18.5
